# A meta-analysis of effects of blended learning on performance, attitude, achievement, and engagement across different countries

**DOI:** 10.3389/fpsyg.2023.1212056

**Published:** 2023-07-12

**Authors:** Wenwen Cao

**Affiliations:** Department of College English Teaching, Qufu Normal University, Rizhao, China

**Keywords:** distance education and online learning, improving classroom teaching, learning communities, mobile learning, pedagogical issues

## Abstract

While this special pandemic period has been seeing an increasing use of blended learning, few studies have meta-analytically reviewed the effectiveness of blended learning in different countries. This meta-analysis summarizes previous studies on blended learning effectiveness in different countries in terms of students' performance, students' attitudes toward blended learning, learning achievement, and student engagement in different countries. Through the meta-analysis via Stata/MP 14.0, it is concluded that blended learning can improve performance, attitude, and achievement in most countries. However, in both China and the USA, blended learning cannot significantly improve student engagement in academic activities. No significant differences were revealed in student performance in the USA between blended and non-blended learning. Future research can extend the research into blended learning to more countries and areas across the world.

## 1. Introduction

This special pandemic time has been witnessing the popularity of blended learning approaches. However, very few studies have summarized blended learning effectiveness in different countries. It is thus meaningful and necessary to examine the effectiveness of blended learning across the world especially during this special time.

### 1.1. Definitions of blended learning

Blended learning, a combination of virtual and physical learning conditions (Al-Qatawneh et al., 2020; Yu et al., 2022), is defined as a learning strategy integrating two different educational models, e.g. distance and traditional learning (Bonk and Graham, 2006). There are three most popular definitions of blended learning (Bonk and Graham, 2012), blending instructional modalities (Yu, 2015; Thomson, 2020; Min and Yu, 2023a), instructional methods (Li and Yu, 2023), and online learning with face-to-face instructional approaches (Young, 2002; Ward and LaBranche, 2003).

### 1.2. Performance

Performance in blended learning can be defined as the measurable outcomes of a student's achievement in both online and offline learning environments (Spanjers et al., 2015). Assessment, evaluation, and analysis can be used to describe performance in blended learning. Some potential ways to assess performance in blended learning include evaluating the quality of student work, analyzing student participation in online discussions, and measuring improvement in learning outcomes from before and after blended learning experiences. The assessment of performance in blended learning should also consider factors such as critical thinking, knowledge delivery, disposition improvement, knowledge and skill improvement, as these have been shown to affect overall success in the blended learning environment.

Most studies positively reported blended learning performances. Performances in this study include the variables: critical thinking skills, knowledge delivery, disposition improvement, knowledge and skill improvement, language use, listening skills, speaking skills, and topic development. Blended learning, outperforming full online learning in the aspects of motivation, attitudes, and satisfaction, can improve nurses' clinical knowledge compared with the traditional learning approach in the UK (McCutcheon et al., 2018). Blended learning can optimize the learning flexibility in terms of time and space, leading to stable learning performance of undergraduates in The Zurich University in Germany (Mueller et al., 2020). It was revealed that both classroom and online learning could enhance American students' learning performance, but the blended learning brought about the largest gain in performance in the USA (Hill et al., 2017). Blended learning could give rise to significantly higher learning performance than e-learning, while the flipped classroom could improve intrinsic motivation and self-efficacy in Can Tho University in Vietnam (Thai et al., 2017).

Numerous studies reported that blended learning was beneficial to language proficiency improvements. Blended learning could greatly improve the reading abilities of children in a kindergarten in the USA (Macaruso et al., 2020). Blended instruction could greatly improve students' English writing abilities in Ankang College, Shanxi China (Zhou, 2018). Blended learning could improve students' English listening and speaking and critical thinking skills, e.g. analysis, inference, evaluation, induction, and deduction in China (Yang et al., 2013). Blended learning could enable Chinese college students to extensively practice with flexible time and space, greatly improving their English reading skills (Yang, 2012).

Blended learning could also enhance high-order abilities such as communication, problem-solving, and reasoning skills. Blending a class video blog into face-to-face instruction could improve language oral proficiency but failed to greatly improve the voluntariness to communication using the target language in China (Liu, 2016). Blended learning could effectively facilitate communication skills and improve learning outcomes of nursing tertiary students in Singapore (Shorey et al., 2018). In the blended learning, Chinese students could discuss with peers, propose meaningful ideas, mutually learn and share, improve group work skills, enhance self-perception, and facilitate reasoning skills (Monteiro and Morrison, 2014). Blended learning could enhance acute stroke patients' competences, e.g., recognition and management in the USA (Lee Gordon et al., 2005).

### 1.3. Attitude

Attitude toward blended learning can be defined as an individual's disposition or perspective regarding the use of a combination of online and traditional face-to-face teaching methods to deliver educational content (Inal and Korkmaz, 2019). This approach provides a flexible and interactive learning environment that allows students to develop their skills and enhance their knowledge through various multimedia channels. One can describe the attitude toward blended learning by examining students' engagement levels, preferences, and motivation toward the use of technology-enhanced learning. Additionally, the effectiveness and efficiency of blended learning can be measured by analyzing students' performance and outcomes in both the physical and virtual learning spaces. The construct attitude in this study includes: self-assessment-cognition, attitudes toward blended learning, blended learning satisfaction, communication skills, self-efficacy, motivation, and confidence.

The majority of learners positively assess the blended learning effectiveness. Blended learning, conducive to students' positive attitude and satisfaction, could improve English listening skills and enhance vocabulary acquisition among junior middle school students in China (Jia et al., 2012). Chinese 11th graders held significantly more positive attitudes toward blended learning than traditional learning (Chang et al., 2014). Singaporean nursing college students had greatly positive attitude toward blended learning, as well as communication skills in the blended context (Shorey et al., 2018). The blended model in active learning classrooms obtained positive evaluation and students held improved attitudes toward physics courses in North Carolina State University in the USA (Beichner et al., 2007). Blended learning could improve nursing students' motivation, satisfaction, and attitude in clinical supervision skills compared with online-only learning in China (Chang et al., 2014).

### 1.4. Achievement

Achievement in blended learning can be defined as the level of success or accomplishment that students attain when participating and completing a blend of online and traditional face-to-face learning activities (Inal and Korkmaz, 2019). This measure of achievement encompasses various learning outcomes, such as improved academic performance, increased engagement, and enhanced critical thinking and problem-solving abilities. In blended learning, the achievement can be assessed through a variety of methods, including graded quizzes and assignments, class participation, peer evaluations, and self-reflection. Additionally, the use of learning analytics and data-driven assessment measures can provide valuable insights into students' progress and provide feedback to instructors for more personalized and effective teaching strategies. Overall, achievement in blended learning is determined by the effectiveness of the instructional design, the quality of the learning materials, and the level of support provided to students throughout the learning process. Achievements in this study include: exam scores, students' knowledge state, writing content relevance, English test scores, actual grades in the academic goal planning assignment, achievement test scores, course grades, level of knowledge, gain in knowledge, student learning outcomes, reading achievements, and academic progress.

Many studies reported that blended learning could contribute to higher learning achievements than traditional approaches. Blended learning could give rise to significantly higher academic achievements than traditional face-to-face learning in Canada (Bazelais and Doleck, 2018). Online learning activities could improve students' academic achievements among undergraduate students in University of Granada in Spain, where influencing factors included attendance rate and students' backgrounds rather than the time they spent on learning (López-Pérez et al., 2013; Min and Yu, 2023b). Blended learning via information and communication technologies could significantly improve learning achievements of mechanical couplings in engineering in Spain (Cortizo et al., 2010). A blended and flipped pedagogical approach could improve learning achievements and learning environment and raise the efficiency of space use in the USA (Baepler et al., 2014).

### 1.5. Engagement

Engagement in blended learning can be defined as the degree to which students are actively involved and invested in the learning process, both online and in-person (George-Walker et al., 2010). This engagement encompasses a wide range of behaviors, including active participation in discussions and group activities, completing assignments and coursework, and seeking out additional learning opportunities outside the formal curriculum. In blended learning, engagement can be fostered through various strategies, such as providing opportunities for students to collaborate and work together, providing feedback on student work, and using interactive multimedia tools and resources to enhance the learning experience. Engagement can also be measured through assessments, such as self-reflection surveys, course evaluations, and quizzes that measure participation and effort. Overall, engagement in blended learning is crucial for promoting student motivation, improving learning outcomes, and creating a supportive learning environment that promotes academic success. The construct engagement in this study includes: time spent learning, student perception of the learning space, and the perception of problem solution.

Most previous studies reported that blended learning could improve learning engagement. Blended learning, encouraging students to engage in learning even after class, could give rise to a significantly higher frequency and level of engagement than the traditional learning in Spain (Pérez-Marín and Pascual-Nieto, 2011). In the technology-oriented blended learning, Chinese freshmen used to spend more time on in-class discussion and writing tasks than the efficiency-oriented group. The interaction was considered an important factor influencing blended learning effectiveness among Chinese freshmen (Yen and Lee, 2011). Undergraduates at Point Loma Nazarene University in the USA spent significantly more time learning in a blended instruction model than in the traditional instruction model (Botts et al., 2018). Blended learning could improve Chinese students' engagement by increasing their learning efficiency and effectiveness (Monteiro and Morrison, 2014).

### 1.6. Contradictory findings

Although blended learning is a popular teaching method, there have been conflicting findings regarding its impact on academic achievement in different countries. For instance, a study conducted in China found that while students believed that blended learning had a positive impact on their achievement, empirical evidence showed no significant improvement (Chang et al., 2014). Similarly, research conducted in Hong Kong China indicated no significant differences in Fashion learning achievements between blended and traditional approaches (Yick et al., 2019). Additionally, research conducted in an American university showed no demonstrable benefits of blended learning in terms of learning outcomes for economics courses (Olitsky and Cosgrove, 2014). Even when blended learning does show some positive benefits, such as an improvement in self-assessment of knowledge gains, this does not necessarily translate into improved academic achievement for Chinese learners (Chang et al., 2014).

Research on blended learning has yielded inconsistent findings regarding its impact on attitude, performance, and engagement. For instance, a study conducted in the United Arab Emirates found no significant differences in attitudes toward blended or traditional approaches, which could be due to either internal or external factors (Al-Qatawneh et al., 2020). Meanwhile, a study in China revealed that efficiency-oriented blended learning significantly improved problem-solving performance among freshmen when compared with hybrid-oriented and technology-oriented groups (Yen and Lee, 2011).

However, research conducted at Point Loma Nazarene University in the USA showed no significant differences between blended and traditional instruction for an upper-division quantitative literacy course, and students also spent less time learning in blended courses (Botts et al., 2018). In a separate study, students in a blended learning course at an undergraduate university in Alberta, Canada had no significant differences in self-efficacy and knowledge scores compared to those using non-blended instruction, despite positively perceiving blended learning (Berga et al., 2021). Overall, the impact of blended learning on attitude, performance, and engagement is inconclusive and may vary depending on factors such as the type of blended learning used and the context in which it is employed.

### 1.7. Research gap

The research gap in previous literature is a lack of comprehensive and up-to-date understanding of the impact of blended learning on performance, attitude, achievement, and engagement in countries with diverse socio-cultural contexts, educational systems and levels, and technological infrastructure. The study aims to address this gap by exploring the magnitude and variability of the effects of blended learning on multiple performance, attitude, achievement, and engagement indicators across different countries, as well as identifying factors that moderate or enhance these effects. The meta-analysis also helps identify gaps in the research literature and suggest future research directions that will inform evidence-based practice and policy in the field of education.

### 1.8. Research questions

Considering the inconsistent findings regarding the influence of blended learning on learner performance, attitude, achievements, and engagement in different countries, we propose four research questions, i.e. (1) Can blended learning positively influence student performance in different countries such as Canada, China, Germany, Spain, The United Arab Emirates, UK, USA, Vietnam, and Singapore? (2) Can blended learning positively influence student attitude in different countries such as Canada, China, Germany, Spain, The United Arab Emirates, UK, USA, Vietnam, and Singapore? (3) Can blended learning positively influence learning achievement in different countries such as Canada, China, Germany, Spain, The United Arab Emirates, UK, USA, Vietnam, and Singapore? (4) Can blended learning positively influence student engagement in different countries such as Canada, China, Germany, Spain, The United Arab Emirates, UK, USA, Vietnam, and Singapore?

## 2. Methods

This meta-analysis was implemented according to the Preferred Reporting Items for Systematic Reviews and Meta-Analyses (PRISMA) (Moher et al., 2009). PRISMA is an evidence-based reporting guideline that provides a checklist of items to include when conducting a systematic review or meta-analysis. PRISMA aims to improve the reporting quality and transparency of systematic reviews and meta-analyses by providing a standardized framework that facilitates the critical appraisal and synthesis of research evidence.

PRISMA consists of a 27-item checklist that covers the title, abstract, introduction, methods, results, discussion, and funding sections of a systematic review or meta-analysis. The checklist includes items such as information on the research question, inclusion and exclusion criteria, search strategy, data extraction, risk of bias assessment, synthesis of results, and limitations of the study. In addition to the checklist, PRISMA also includes a flowchart that illustrates the process of selecting studies for inclusion in the systematic review or meta-analysis. The flowchart outlines the number of studies that were initially identified, the number that were excluded based on inclusion and exclusion criteria, and the number that were finally included in the review. PRISMA has become a widely adopted reporting guideline in the field of healthcare research and has been found to improve the quality and transparency of systematic reviews and meta-analyses. Its implementation has also allowed for greater comparability and synthesis of research evidence, which ultimately supports evidence-based decision making in healthcare.

### 2.2. Eligibility criteria

The studies will be considered eligible and included if they (1) focus on the effect of blended learning on performance, student attitude, achievements, and engagement in different countries; (2) are highly evaluated using University of West England Framework for Critically Appraising Research Articles (Moule et al., 2003); (3) can provide enough data for a meta-analysis; (4) divide the participants into both control and experimental groups for a comparative analysis between blended learning and non-blended learning; and (5) are written in the standard English language.

The studies will be considered ineligible and excluded if they (1) focus on blended learning technologies themselves rather than blended learning effect; (2) cannot provide enough data for a meta-analysis even after we correspond with the authors; (3) are not written in English; or (4) they are poorly evaluated using University of West England Framework for Critically Appraising Research Articles (Moule et al., 2003).

### 2.3. Data sources and search strategy

**Figure 1:**
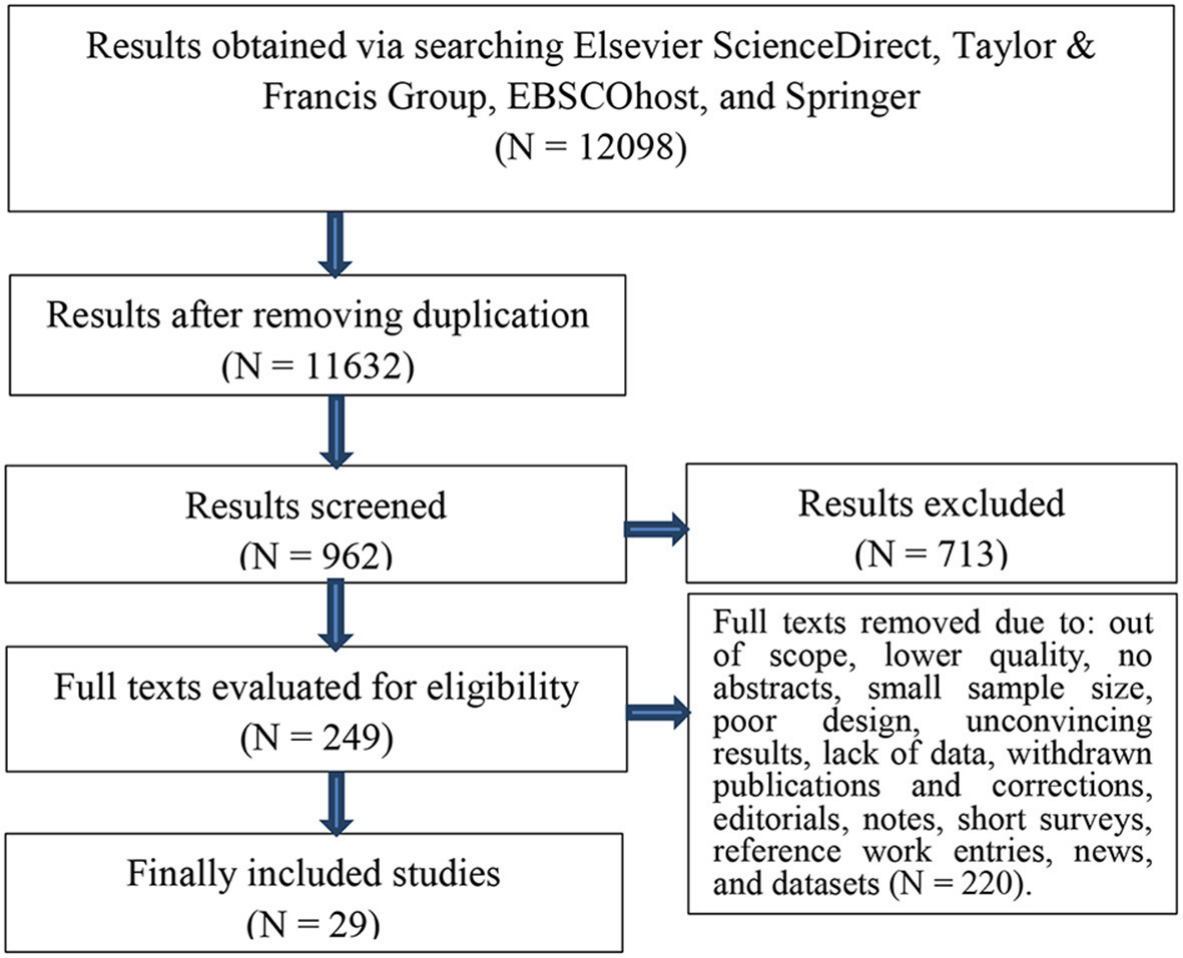
A flowchart of literature inclusion.

Based on the PRISMA flow (Figure 1), we conducted the inclusion and exclusion process. To maximize the number of data included, we searched the databases from their inception until February 26, 2023 without time limitation. We entered keywords and index terms, e.g., *blended learning, performance, attitude, achievements*, and *engagement, different countries*, into different databases according to their specific syntactical rules. We obtained 12,098 results by searching four online databases, i.e., Elsevier ScienceDirect, Taylor and Francis Group, EBSCOhost, and Springer. Then we entered the results into ENDNOTE X8 (Thomson Reuters, New York, USA) to remove those duplicated. Then we invited two researchers to double-check whether or not the results are related to the study by screening the titles and abstracts. After this, they conducted the evaluation of eligibility of the results.

Finally, two researchers met to decide on the included studies for the meta-analysis. They discussed different selected studies and negotiated to address the disputes. Those selected by both of them were directly included in the meta-analysis. A third reviewer will be invited to finally determine the finally selected studies in case two researchers cannot reach an agreement on the inclusion of any study.

### 2.4. Evaluation of included studies

We evaluated the full texts via University of West England Framework for Critically Appraising Research Articles (Moule et al., 2003). This framework evaluates the research articles based on five sections, i.e., the introduction, the methods, ethics, the results/findings, and the conclusions. Each section has detailed criteria for evaluation. For the method section, we use different criteria for different methods, e.g., qualitative or quantitative research. We also use specific criteria to evaluate data collection and analysis. We finally included 29 results for the meta-analysis (Table 1). The inter-rater consistency reaches a satisfactory level (Cohen's kappa coefficien*t* = 0.83). This indicates that two researchers mostly selected the same studies or generally reached an agreement on most of the selected studies.

**Table 1:** The included studies for the meta-analysis.

**N**	**Author/year**	**Source**	**Subgroup**	**Country**
1	Al-Qatawneh et al. (2020)	Springer	Achievement and attitude	The United Arab Emirates
2	Baepler et al. (2014)	Elsevier	Achievement, attitude, engagement, and performance	USA
3	Bazelais and Doleck (2018)	Springer	Achievement	Canada
4	Botts et al. (2018)	Taylor and Francis	Engagement	USA
5	Chang et al. (2014)	EBSCOhost	Achievement, attitude, and performance	China
6	Cortizo et al. (2010)	Elsevier	Achievement	Spain
7	Lee Gordon et al. (2005)	EBSCOhost	Achievement	USA
8	Hill et al. (2017)	EBSCOhost	Achievement	USA
9	Jia et al. (2012)	Elsevier	Achievement	China
10	Liu (2016)	Elsevier	Achievement and performance	China
11	López-Pérez et al. (2013)	Springer	Achievement	Spain
12	Macaruso et al. (2020)	Springer	Achievement	USA
13	McCarthy et al. (2020)	Taylor and Francis	Achievement	USA
14	McCutcheon et al. (2018)	Elsevier	Achievement and attitude	UK
15	Monteiro and Morrison (2014)	Taylor and Francis	Engagement	China
16	Mueller et al. (2020)	Taylor and Francis	Achievement	Germany
17	Olitsky and Cosgrove (2014)	Elsevier	Achievement and performance	USA
18	Pérez-Marín and Pascual-Nieto (2011)	Springer	Achievement	Spain
19	Shorey et al. (2018)	Elsevier	Attitude	Singapore
20	Thai et al. (2017)	Elsevier	Achievement, attitude, and performance	Vietnam
21	Yang et al. (2013)	Elsevier	Performance	China
22	Yang (2012)	Taylor and Francis	Performance	China
23	Yen and Lee (2011)	Elsevier	Performance	China
24	Yen and Lee (2011)	Elsevier	Achievement	China
25	Yick et al. (2019)	Taylor and Francis	Achievement	China
26	Zhou (2018)	EBSCOhost	Achievement	China
27	Huang et al. (2022)	Elsevier	Performance	China
28	Rattanasak (2023)	MERT BASTAS PUBLISHING CO	Performance	Thailand
29	Zhou (2023)	Routledge	Achievement	China

### 2.5. Data extraction

Two professor solicited specific information such as author, publication year, and the source of the literature. We also collected enough data for the meta-analysis such as means, standard deviations, and numbers of participants for both control and experimental groups. For convenience of analysis, we classified the findings into *performance, attitude, achievements*, and *engagement*, followed by the countries where the studies were conducted. The selected were implemented in various countries across the world such as China, the United Arab Emirates, Canada, the USA, Spain, Germany, Singapore, and Vietnam. We will compare different effects of blended learning in these countries. Similarly, both researchers would meet up to discuss different results of data extraction and a third reviewer would be invited to decide the final data if any disagreement occurred between two researchers. The inter-rater consistency also reaches a satisfactory level (Cohen's kappa coefficien*t* = 0.81).

### 2.6. Statistical analysis

We meta-analytically examined the data using Stata/MP 14.0. After entering data such as numbers of participants, means, and standard deviations for both groups into Stata/MP 14.0, forest plots will be drawn. We calculated *standardized mean difference* (SMD or Cohen *d*) (Cohen, 1988) indicating the effect sizes, *weight* indicating the degree of the influence on pooled results, and 95% confidence interval indicating the study reliability. Cohen *d* is produced through dividing the mean difference between both groups by the pooled standard deviation of both groups (Sedgwick and Marston, 2013). The formula is: Cohen *d* = (M2-M1)/Pooled SD, where M1 indicates the mean of the control group) and M2 indicates the mean of the experimental group. The effect size will be deemed *very small* if *d* approximates 0.1, *small* if *d* approximates 0.2, *medium* if *d* approximates 0.5, *large* if *d* approximates 0.8, *very large* if *d* approximates 1.2, *huge* if *d* approximates 2.0 (Sawilowsky, 2009).

To determine whether a random-effect or a fixed-effect model could be adopted, we also tested the heterogeneity of the effect sizes using I^2^ and *p* values. The formula to calculate I^2^ is: I^2^ = [(Q-df)/Q] × 100%, where Q indicates the Chi-squared statistics and df means the degree of freedom (Higgins and Thompson, 2002; Higgins et al., 2003). This indicates the degree of percentage of the variability in effect sizes caused by heterogeneity or random errors. According to Higgins and Green (2021), the heterogeneity will be considered *unimportant* in case I^2^ ranges from 0 to 40%, *moderate* in case I^2^ ranges from 30 to 60%, *substantial* in case I^2^ ranges from 50 to 90%, and *considerable* in case I^2^ ranges from 75 to 100%. Generally, if I^2^ is larger than 50% (*p* < 0.05), we will adopt a random-effect model to conduct the meta-analysis, and if I^2^ is smaller than 50% (*p* > 0.05), we will use a fixed-effect model to run the meta-analysis. The influence analysis program will be used to run the sensitivity analysis. Both Begg and Mazumdar (1994) and Egger et al. (1997) tests will be used to test the publication bias.

## 3. Results

### 3.1. Tests of publication bias

Publication bias in a meta-analysis refers to the systematic exclusion or underrepresentation of studies with negative or non-significant results from the analysis. This occurs when studies that report significant or positive findings are more likely to be published in academic journals, while studies with null or negative findings are less likely to be published. As a result, when a meta-analysis is conducted, there is a risk that it may overestimate the effects of an intervention or treatment due to the missing or underrepresented data. This bias can lead to incorrect conclusions and incorrect recommendations for clinical practice. Publication bias can also occur for a variety of reasons, including the behavior of authors, reviewers, and editors, as well as the funding source of the studies. To address publication bias in a meta-analysis, researchers can use methods such as funnel plots, which help identify any asymmetry in the distribution of studies. They can also conduct sensitivity analyses to examine the impact of potential studies that may be missing. Additionally, researchers can conduct a comprehensive search for all studies, including unpublished studies, to reduce the risk of publication bias.

**Figure 2:**
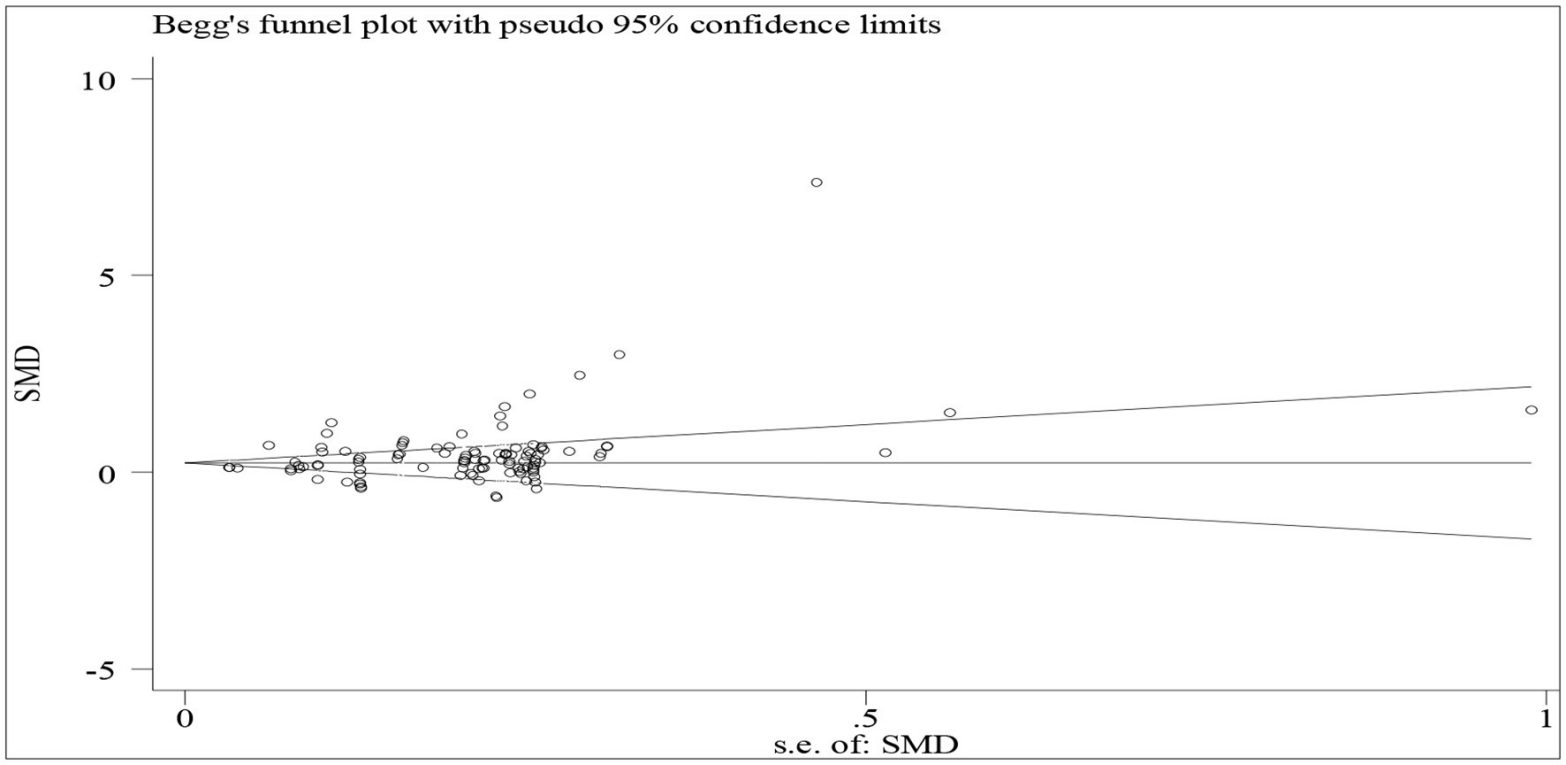
A funnel plot of tests for publication bias.

To test the publication bias, we firstly entered data, e.g., means, standard deviation, and numbers of participants across both groups, into Stata/MP 14.0 to run the meta-analysis. Then, we obtained effect sizes (ES) and standard errors of effect sizes (seES) for the test of publication bias. We tested the publication bias by entering “ES, seES” into Stata/MP 14.0, leading to a funnel plot (Figure 2) and related data. A dot indicates an individual study, and the middle line is the no-effect line. If the dots are symmetrically distributed along both sides of the no-effect line, there will be an absence of publication bias. On the contrary, the asymmetrical distribution indicates the presence of publication bias. As shown in Figure 2, it is hard to conclude that the dots are symmetrically distributed, indicating the presence of publication bias. Both Begg's (Q = 1016, S.D. = 381.89, *z* = 2.66, *p* = 0.008) and Egger's tests (Coefficien*t* = 1.55, S.E. = 0.48, *t* = 3.25, *p* = 0.002, 95% CI = 0.60~2.48) also indicate the presence of publication bias.

### 3.2. A sensitivity analysis

We conducted a sensitivity analysis to test the reliability and stability of the obtained effect sizes using the program “metan-based influence analysis”. To retrieve the result, we entered the data such as means, standard deviations, and numbers of both groups into Stata/MP 14.0. We adopted a random-effect model to conduct the sensitivity analysis due to the high degree of percentage of variability caused by heterogeneity (Q = 1053.01, I^2^ = 89.7%, *z* = 8.88, *p* < 0.01).

**Figure 3:**
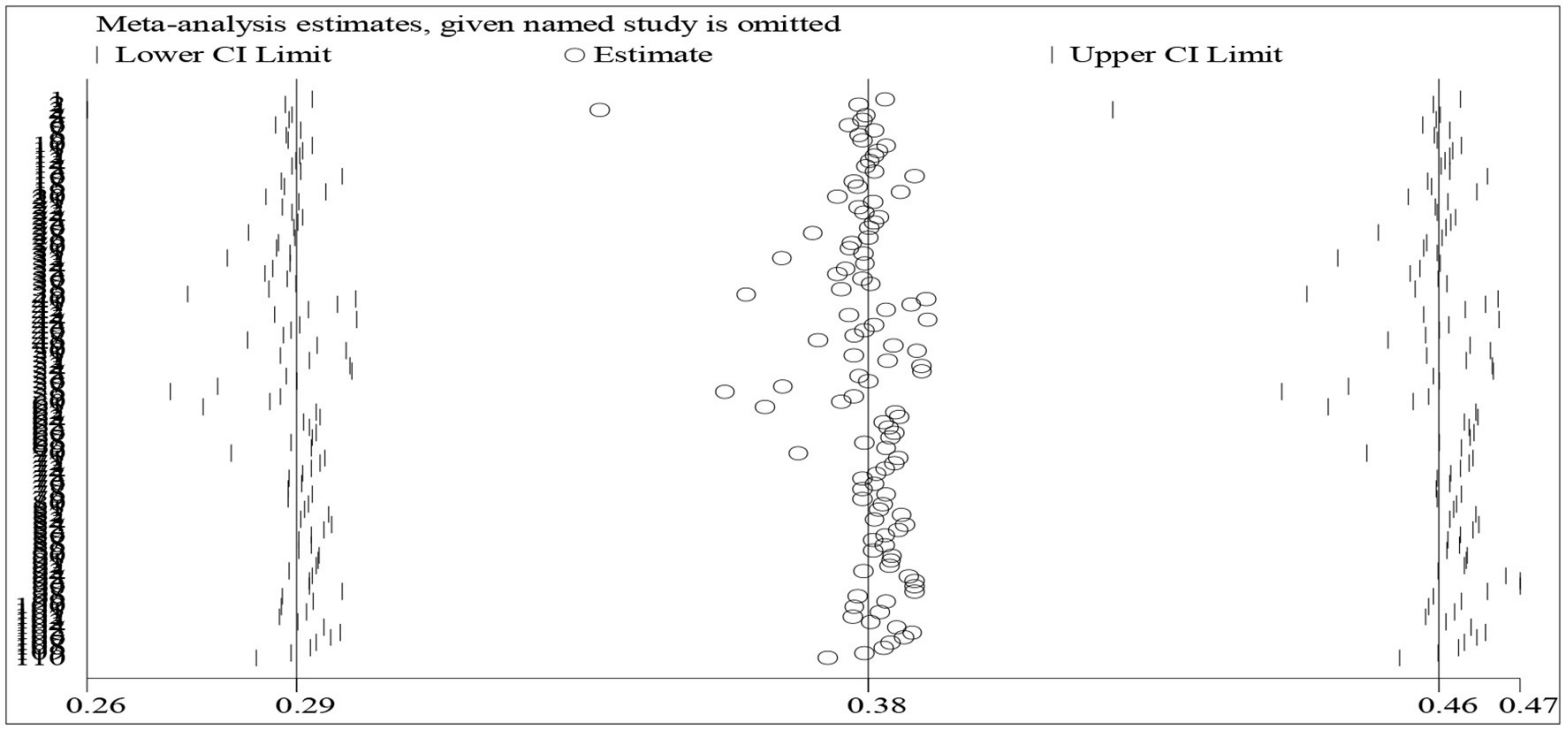
A plot of results of the sensitivity analysis.

Unstable ES estimates often lead to skewed distribution and are frequently located beyond the lower and upper bounds of 95% confidence intervals (Borenstein et al., 2009). It is thus a must to identify whether there is any estimate located beyond the scope of 95% confidence intervals (Borenstein et al., 2009). As shown in Figure 3, a dot indicates an estimated effect size of an individual study. All the effect sizes are located within the low and upper bounds of 95% confidence intervals. This indicates that there are no unstable ES estimates. We, therefore, conclude that the meta-analysis results are stable.

### 3.3. Can blended learning positively influence student performance in different countries?

**Figure 4:**
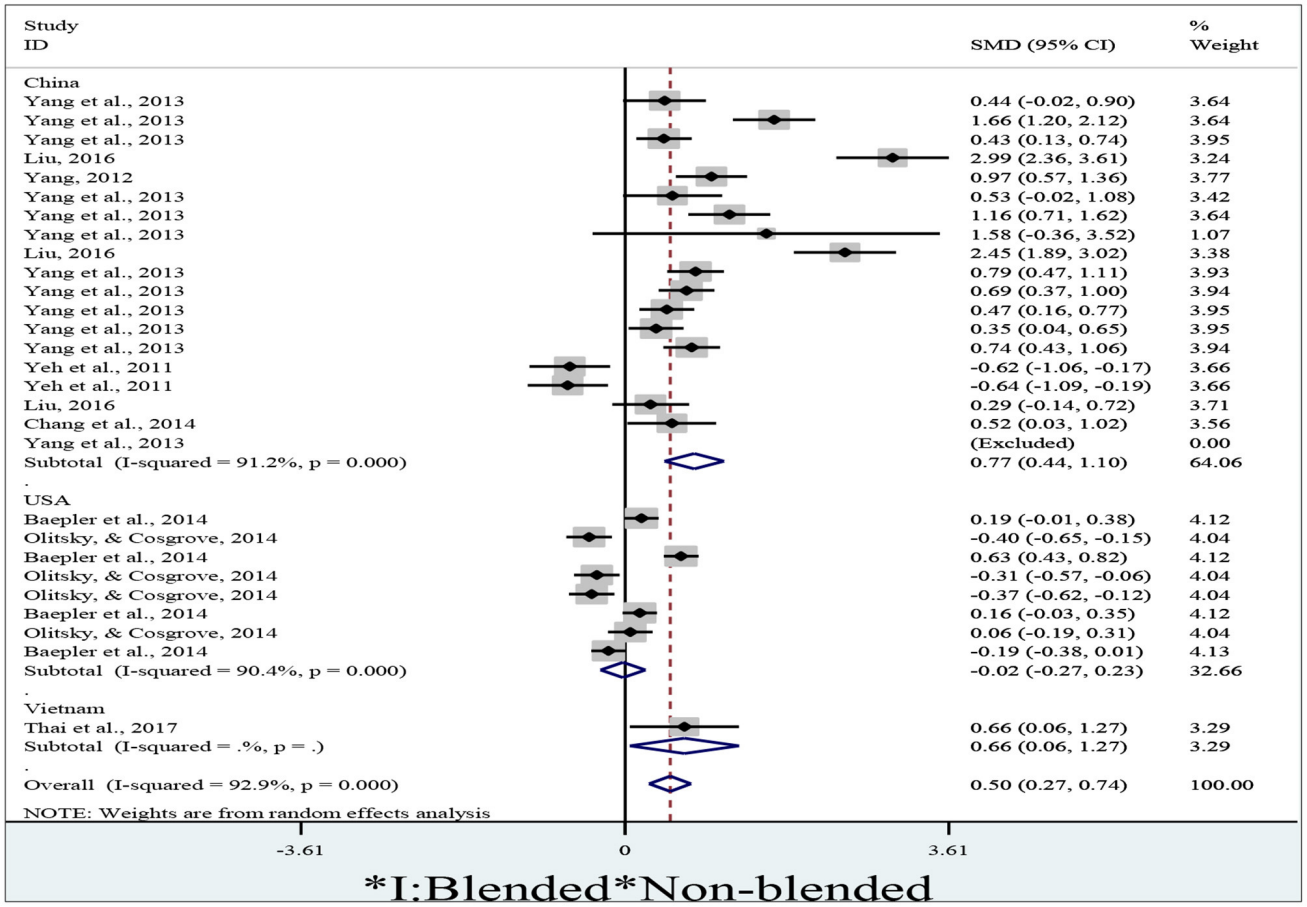
A forest plot of student performance in different countries.

To determine student performance in blended and non-blended learning modes in different countries, we retrieved 27 effect sizes from different countries, where 18 effect sizes sourced from China, 8 from the USA, and 1 from Vietnam. We failed to obtain an effect size from a study (Yang et al., 2013) because one of the standard deviation values is zero. We obtained meta-analytical data and a forest plot (Figure 4) after entering means, standard deviations and, numbers across both groups into Stata/MP 14.0 to run the meta-analysis by the variable *country*.

Figure 4 is a forest plot, a type of graph commonly used in meta-analyses to display the results of multiple studies on a particular topic. In the case of student performance in different countries, a forest plot will provide a visualization of the main findings of a meta-analysis that looked at academic performance of students in different countries. In Figure 4, each study is represented by a horizontal line called a square. The size of the square represents the weight or sample size of the study that contributes to the overall analysis. The position of the square on the vertical axis represents the effect size of the study. In the context of student performance, the effect size may be represented as standardized test scores or other measures of academic achievement.

The forest plot also includes a vertical line (often called a diamond) that represents the overall effect size of the meta-analysis. The width of the diamond reflects the confidence interval of the effect size. In the case of the student performance meta-analysis, each square in the forest plot will represent a study that measured academic achievement in different countries. The position of each square on the vertical axis will represent the effect size or standardized test scores in that country. By analyzing the forest plot, researchers can identify which countries have higher or lower academic performance on average, and compare the effect sizes of different studies to assess the consistency of the results across the studies in the meta-analysis. Forest plots can help researchers and policymakers understand how countries compare to each other in terms of academic performance and make more informed decisions about educational policy.

As shown in Figure 4, the diamonds at the bottom indicate the pooled results. In the left-most column are displayed the author names and publication years, followed by a middle line with numerous boxes. The middle line is referred to as a no-effect line because if a diamond crosses it, the result will be considered insignificant. A box, integrated with a horizontal line and a dot, indicates an individual study. The length of the horizontal line is negatively related to the reliability of the study. The dot indicates the SMD. On the right are displayed the statistics of SMDs (Cohen *d*) and 95% confidence intervals after them. The right-most column shows the weights indicating the influence of effect sizes on the pooled result.

We adopted a random-effect model to run the meta-analysis of the data sourcing from China (I^2^ = 91.2%, *p* < 0.01), the USA (I^2^ = 90.4%, *p* < 0.01) and Vietnam (a single study) due to a generally high degree of percentage of variability caused by heterogeneity (I^2^ = 92.9%, *p* < 0.01).

As for the meta-analysis of data sourcing from China and Vietnam, the diamonds are located to the right of the no-effect line. This indicates that student performances in the blended learning context in China (*d* = 0.77, 95% CI = 0.44~1.10, *z* = 4.59, *p* < 0.01) and Vietnam (*d* = 0.66, 95% CI = 0.06~1.27, *z* = 2.14, *p* = 0.032) are significantly higher than the non-blended. However, the diamond retrieved from the data sourcing from the USA crossed the no-effect line, indicating that student performance in the blended learning context in the USA (*d* = −0.02, 95% CI = −0.27~0.23, *z* = 0.19, *p* = 0.853) is not significantly higher than the non-blended. The overall results indicate that the blended learning can lead to significantly (*d* = 0.50, 95% CI = 0.27~0.74, *z* = 4.24, *p* < 0.01) higher student performance than the non-blended since the diamond is located to the right of the no-effect line. In general, we believe that blended learning could positively influence student performance in different countries.

### 3.4. Can blended learning positively influence student attitude in different countries?

To determine the differences in student attitudes between blended and non-blended learning in different countries, we obtained totally 11 effect sizes from the studies sourcing from the United Arab Emirates, China, Singapore, Vietnam, the UK, and the USA. We adopted a random-effect model to conduct the meta-analysis due to the high degree of percentage of variability of the effects sizes sourcing from different countries caused by heterogeneity (I^2^ = 76.9%, *p* < 0.01).

**Figure 5:**
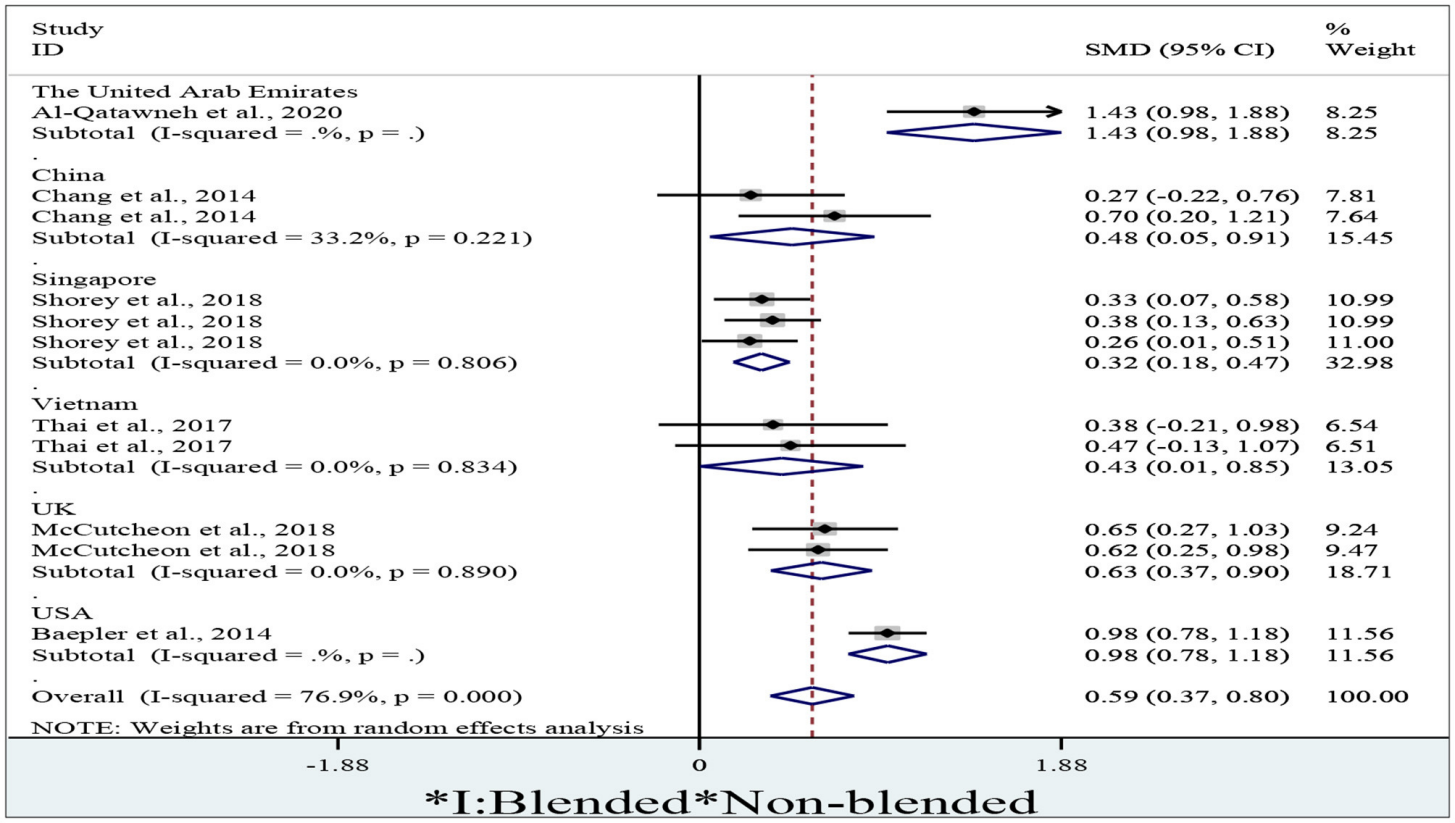
A forest plot of student attitude in different countries.

Figure 5, a forest plot of student attitude toward blended learning in different countries, will likewise show a graphical representation of the results of a meta-analysis of studies on this topic. Each study in the meta-analysis will be represented by a square on the graph, with the size of the square representing the sample size of the study, and the position on the vertical axis representing the effect size of the study (i.e., the average student attitude toward blended learning). The forest plot will also include a vertical line (often called a diamond) that shows the overall effect size of the meta-analysis, as well as its confidence interval. The forest plot will allow researchers to compare the effect sizes of different studies across countries, providing important insights into how student attitudes toward blended learning vary across different educational contexts. Researchers and policymakers can use these insights to identify which countries have more positive or negative attitudes toward blended learning, and to guide their decisions about educational policy and instructional design.

As shown in Figure 5, students present significantly more positive attitudes in the blended context than in the non-blended in the United Arab Emirates (*d* = 1.43, 95% CI = 0.98~1.88, *z* = 6.17, *p* < 0.01), China (*d* = 0.48, 95% CI = 0.05~0.91, *z* = 2.20, *p* = 0.027), Singapore (*d* = 0.32, 95% CI = 0.18~0.47, *z* = 4.37, *p* < 0.01), Vietnam (*d* = 0.43, 95% CI = 0.01~0.85, *z* = 1.98, *p* = 0.047), the UK (*d* = 0.63, 95% CI = 0.37~0.90, *z* = 4.73, *p* < 0.01), and the USA (*d* = 0.98, 95% CI = 0.78~1.18, *z* = 9.41, *p* < 0.01) since their diamonds are all located to the right of the no-effect line without crossing it. The overall result also indicates that blended learning could give rise to significantly more positive student attitude toward blended learning (*d* = 0.59, 95% CI = 0.37~0.80, *z* = 5.28, *p* < 0.01).

### 3.5. Can blended learning positively influence learning achievement in different countries?

**Figure 6:**
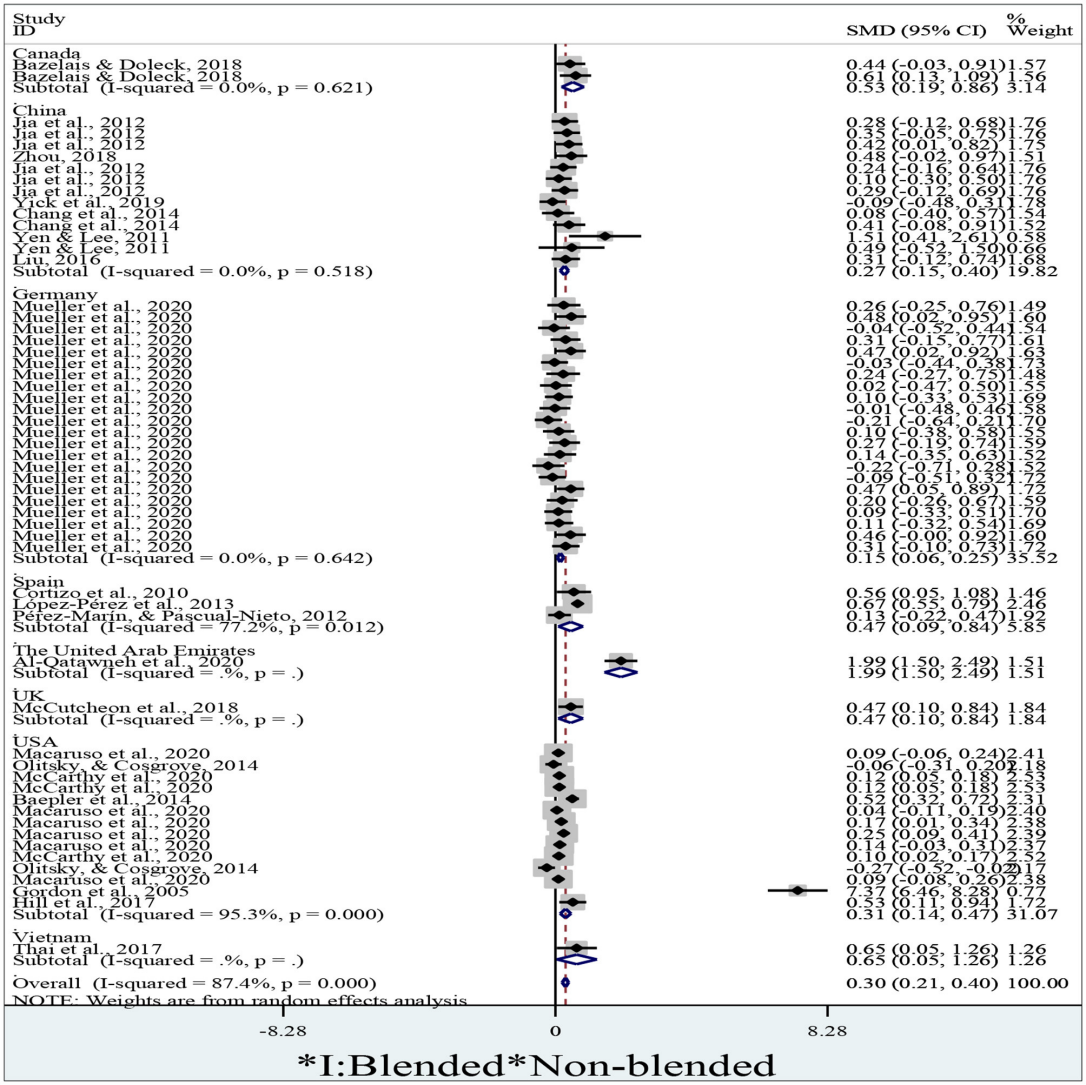
A forest plot of students' achievements in different countries.

To identify students' achievements of blended learning in different countries, we extracted 57 effect sizes, where 2 of them sourced from Canada, 13 from China, 22 from Germany, 3 from Spain, 1 from the United Arab Emirates, 1 from the UK, 1 from Vietnam, 14 from the USA. We adopted a random-effect model to implement the meta-analysis due to the high degree of percentage of variability caused by heterogeneity (I^2^ = 87.4%, *p* < 0.01). We entered means, standard deviations, and numbers of participants across both groups into Stata/MP 14.0, then we obtained a forest plot after running the meta-analytical program by the variable *country* (Figure 6).

Figure 6, a forest plot of students' achievements in different countries in a meta-analysis, will visually represent the results of multiple studies that examine the academic performance of students in various countries. Each study included in the analysis will be represented by a square on the graph, where the size of the square represents the sample size of the study, and the position on the vertical axis shows the effect size (i.e., the average achievement score) of the study. The graph will also include a horizontal line (often called a diamond) that denotes the overall effect size estimate of the meta-analysis, along with its confidence interval. A forest plot of this nature will be useful in comparing the academic performance of students across different countries and regions. Researchers and policymakers can use these insights to gain a better understanding of how student achievement varies across borders, and to identify those countries with higher or lower student achievement scores. This information can guide education policy decisions, such as the allocation of resources and the implementation of targeted interventions to improve student performance.

The pooled diamond at the bottom is located to the right of the no-effect line without crossing it. We thus conclude that the students' overall achievement in the blended learning context is significantly larger than that in the non-blended learning context (*d* = 0.30, 95% CI = 0.21~0.40, *z* = 6.24, *p* < 0.01). No diamonds, the pooled results, for different countries cross the no-effect middle line and all of them are located to the right of it. Consequently, students' blended learning achievements also significantly surpass the non-blended in Canada (*d* = 0.53, 95% CI = 0.19~0.86, *z* = 3.07, *p* = 0.002), China (*d* = 0.27, 95% CI = 0.15~0.40, *z* = 4.25, *p* < 0.01), Germany (*d* = 0.15, 95% CI = 0.06~0.25, *z* = 3.13, *p* = 0.002), Spain (*d* = 0.47, 95% CI = 0.09~0.84, *z* = 2.44, *p* = 0.015), the United Arab Emirates (*d* = 1.99, 95% CI = 1.50~2.49, *z* = 7.87, *p* < 0.01), the UK (*d* = 0.47, 95% CI = 0.10~0.84, *z* = 2.46, *p* = 0.014), Vietnam (*d* = 0.65, 95% CI = 0.05~1.26, *z* = 2.11, *p* = 0.035), and the USA(*d* = 0.31, 95% CI = 0.14~0.47, *z* = 3.62, *p* < 0.01). We, therefore, believe that blended learning could positively influence learning achievement in different countries.

### 3.6. Can blended learning positively influence student engagement in different countries?

**Figure 7:**
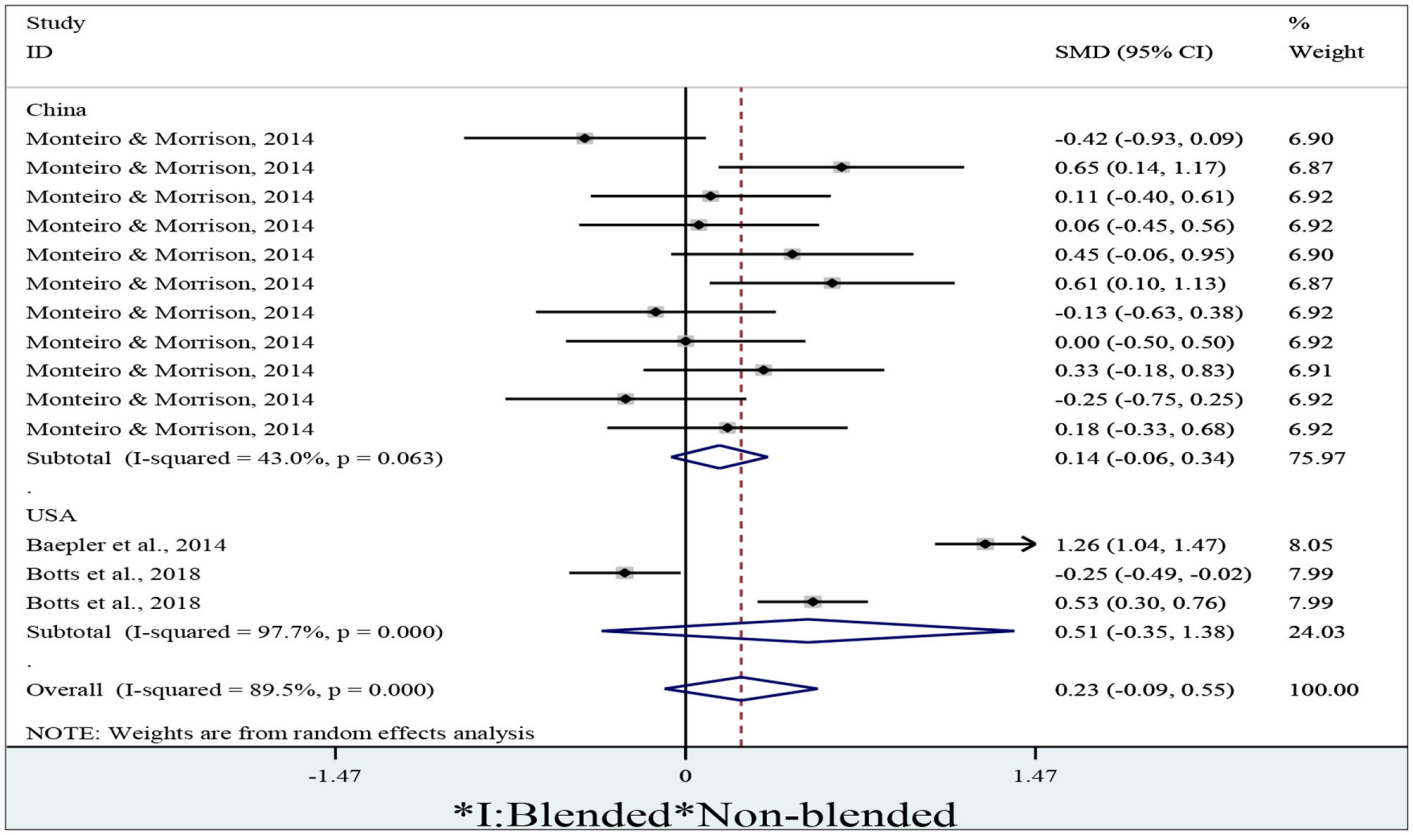
A forest plot of students' engagement in different countries.

To identify whether the blended approach could improve student engagement in learning, we extracted 14 effect sizes, where 3 of them sourced from the USA, and 11 from China. We adopted a random-effect model to conduct the meta-analysis due to a high degree of percentage of variability of effect sizes caused by heterogeneity (I^2^ = 89.5%, *p* < 0.01). After entering means, standard deviations, and numbers of participants of both groups into Stata/MP 14.0, we obtain a forest plot (Figure 7) from the meta-analysis by the variable *country*.

Figure 7, a forest plot of students' engagement in different countries in a meta-analysis, will visually represent the results of multiple studies that examine the levels of engagement of students in different countries. Each study included in the analysis will be represented by a square on the graph, where the size of the square represents the sample size of the study, and the position on the vertical axis shows the effect size (i.e., the average level of engagement) of the study. The graph will also include a horizontal line (often called a diamond) that denotes the overall effect size estimate of the meta-analysis, along with its confidence interval. A forest plot of this nature will be useful in comparing the levels of engagement of students across different countries and regions. It can also be used to identify factors that contribute to higher or lower levels of engagement, such as teaching methods, learning experiences, and cultural factors. This information can guide policymakers and educators in developing interventions that promote higher levels of student engagement, leading to better academic performance and overall wellbeing.

As shown in Figure 7, the diamonds obtained from the meta-analysis of data sourcing from both China and the USA cross the no-effect middle line. The diamond for the overall result also crosses the no-effect middle line. We thus conclude that there are significant differences in student engagement between blended and non-blended learning in both China (*d* = 0.14, 95% CI = −0.06~0.34, *z* = 1.38, *p* = 0.169), the USA (*d* = 0.51, 95% CI = −0.35~1.38, *z* = 1.16, *p* = 0.245), and the overall results (*d* = 0.23, 95% CI = −0.09~0.55, *z* = 1.42, *p* = 0.156). Therefore, we believe that blended learning could not positively influence student engagement in different countries.

## 4. Discussion

Blended learning has been found to have a positive impact on student outcomes such as performance, attitude, and learning achievement in various countries. This conclusion is supported by the results of previous studies which mostly demonstrate the positive effects of blended learning on these outcomes (Yen and Lee, 2011; Chang et al., 2014). However, it is worth noting that some studies have reported negative effects on student engagement when using a blended learning approach (Botts et al., 2018).

The enhanced student performance observed in blended learning can be attributed to several factors. One crucial factor is that students receive instruction in both physical and online environments. In the classroom setting, students are able to ask questions and interact with their peers and teachers for academic issues. Additionally, they receive more individualized attention from their instructors which encourages them to be more engaged in the learning process. By being asked to answer questions and focus on the course material, students are able to improve their performance (Huang et al., 2022).

Blended learning also provides students with greater access to online resources that they can use to supplement their learning. These resources can include multimedia content, virtual simulations, and interactive quizzes. Consequently, students are able to explore topics more deeply and revisit information whenever they need to. Furthermore, they are able to learn at their own pace and in a location of their choosing, which reduces the burden of travel time and carrying heavy books (Yu and Yi, 2020).

In addition to improving performance and achievement, blended learning has been found to positively impact student attitudes toward learning. The convenience that blended learning offers is a key factor in generating a favorable attitude among students. Students can access learning materials at any time from their device, enabling them to learn wherever they are and at their own pace. This is in contrast to traditional classroom learning where students have to carry heavy textbooks and are restricted to learning only during scheduled class times (Yu et al., 2019).

Moreover, blended learning facilitates online interactions among students, allowing them to work collaboratively, share opinions, and create a supportive learning environment. This enhances the effectiveness of their learning and promotes a positive attitude toward the blended learning approach. The power of the Internet is fully utilized in blended learning to overcome the limitations of physical classrooms, thereby creating a flexible and engaging learning environment that better meets the needs of students. In addition, blended learning integrates formal instruction with informal learning. As a result, students can benefit from both learning contexts and engage with learning materials from various sources. This approach provides them with more learning resources and diverse learning experiences that enrich their knowledge and broaden their perspectives. The seamless linking of formal instruction and informal learning contributes to a student-centered approach to blended learning that enhances attitudes toward learning.

Blended learning has been shown to be an effective method for achieving academic success. The approach requires instructors to make learning materials available via the Internet or a learning platform, enabling students to access content and information at any time. Furthermore, experts and instructors' contact information is made readily available online, allowing learners to seek further assistance or explore topics in more depth. The benefit of face-to-face interaction in a physical classroom can also be incorporated into the approach. Online courses play a vital role in transmitting learning materials and facilitating communication among learners. This fosters a deeper understanding of course content and strengthens overall comprehension (Yu and Wang, 2016). Moreover, blended learning encourages students to construct their own knowledge and share their insights with others through the Internet. This process encourages creativity and the exchange of ideas, leading to enhanced learning outcomes for students.

Despite the advantages of blended learning, there are potential challenges to learner engagement. Technical issues with online learning platforms can impede students' progress, including unstable system environments, slow computer speeds, and software compatibility problems. Poorly designed menus and interfaces can also cause frustration, as can slow or unreliable internet connections, which limit the ability to multi-task and may ultimately reduce students' enthusiasm for blended learning. These challenges can erode students' confidence in their computer skills, leading to a decline in overall engagement and, in extreme cases, abandonment of the blended learning approach (Sun and Rueda, 2012).

Blended learning can be impacted by the varied backgrounds and experiences of learners, influenced by factors such as geographic location, family background, and prior education. Educational institutions must recognize this and provide training programs to improve students' online technology skills (Bernard et al., 2014). Recorded videos with detailed operation instructions can be especially helpful in bridging gaps in technology skills among students. To ensure that learners from diverse backgrounds can effectively adapt to the blended learning environment, educational departments should regularly implement training programs. This will help students develop the necessary skills to succeed in online learning and overcome any barriers they may face due to their backgrounds and experiences.

Effective curriculum design is essential for increasing engagement in blended learning (Vaughan, 2007). Teachers should leverage the benefits of online learning and integrate them into traditional classroom teaching. Curriculum design must be based on learners' needs and include visual and aural stimuli to enhance engagement. In addition, teachers should work to improve students' self-efficacy, spark their interest in learning, and motivate them to keep engaging with the material to increase their knowledge. Students' strong sense of self-efficacy and satisfaction can drive their voluntary participation in blended instruction and make them more likely to stay engaged throughout the program. One way to improve engagement is to use MOOCs (Massive Open Online Courses) to blend face-to-face courses (de Moura et al., 2021). This approach can increase flexibility and make curriculum content available to learners at any time or place. Ultimately, effective curriculum design, combined with the use of innovative methods like MOOCs, can help increase engagement in blended learning programs and lead to better learning outcomes for students.

## 5. Conclusion

### 5.1. Major findings

In this meta-analysis, researchers explored the effectiveness of blended learning compared to traditional, non-blended approaches in various countries. The study focused on key areas such as performance, attitude, achievement, and engagement. Overall, the findings suggested that blended learning can lead to improved performance, attitude, and achievement in many countries. However, when it came to student engagement in academic activities, results from both China and the USA were not significantly different between blended and non-blended learning approaches. Interestingly, in the USA, there were no significant differences in student performance between blended and non-blended learning. While blended learning can produce positive results in many areas, it may not be the best fit for all types of students or settings. Therefore, it is important for educators to carefully consider the needs of their students and the learning environment when determining whether or not to implement a blended approach.

### 5.2. Limitations

There are several limitations to this study. Firstly, the meta-analysis cannot include all the publications and non-published works due to the limitation of library sources. Secondly, Both Begg's and Egger's tests indicate the presence of publication bias. Thirdly, we cannot completely retrieve the identified research due to various reasons.

### 5.3. Future research directions

Effective teaching in both blended and non-blended learning environments requires specific instruction that encourages collaboration and practice. This instruction can help students understand the benefits and challenges associated with each approach, ultimately leading to improved learning outcomes (Monteiro and Morrison, 2014). Although blended learning has become increasingly popular in the twenty first century, particularly during the COVID-19 pandemic, research on its effectiveness across different countries is still limited. Future studies can expand this research to examine blended learning approaches in other countries and regions around the world. By exploring the effectiveness of blended learning in a variety of contexts, educators can gain valuable insights and improve teaching practices to better meet the needs of their students. It is essential to conduct this research to ensure that students receive the best possible educational experience regardless of their location or circumstances.

## Data availability statement

The original contributions presented in the study are included in the article/Supplementary material, further inquiries can be directed to the corresponding author.

## Author contributions

WC: designed, wrote, revised, and proofread this article.
